# Crosstalk between purinergic receptors and lipid mediators in leishmaniasis

**DOI:** 10.1186/s13071-016-1781-1

**Published:** 2016-09-05

**Authors:** Mariana M. Chaves, Cláudio Canetti, Robson Coutinho-Silva

**Affiliations:** 1 Laboratory of Immunophysiology, Biophysics Institute Carlos Chagas Filho, Federal University of Rio de Janeiro, Rio de Janeiro, RJ 21941-902 Brazil; 2 Laboratory of Inflammation, Biophysics Institute Carlos Chagas Filho, Federal University of Rio de Janeiro, Rio de Janeiro, RJ 21941-902 Brazil; 3 National Institute of Translational Research in Health and Environment in the Amazon Region, Biophysics Institute Carlos Chagas Filho, Federal University of Rio de Janeiro, Rio de Janeiro, RJ 21941-902 Brazil

## Abstract

Leishmaniasis is a neglected tropical disease affecting millions of people around the world caused by organisms of the genus *Leishmania*. Parasite escape mechanisms of the immune system confer the possibility of resistance and dissemination of the disease. A group of molecules that has become a target for *Leishmania* survival strategies are lipid mediators. Among them, leukotriene B_4_ (LTB_4_) has been described as a pro-inflammatory molecule capable of activating cells of the immune system to combat *Leishmania*. In an opposite way, prostaglandin E_2_ (PGE_2_) is a lipid mediator described as a deactivator of macrophages and neutrophils. The balance of these two molecules can be generated by extracellular nucleotides, such as adenosine 5'-triphosphate (ATP) and adenosine (Ado), which activate the purinergic receptors system. Herein, we discuss the role of extracellular nucleotides and the resulting balance of LTB_4_ and PGE_2_ in *Leishmania* fate, survival or death.

## Review

### Background

The protozoan parasites of the genus *Leishmania* cause a broad range of human diseases called leishmaniasis. Leishmaniasis is a neglected disease of tropical and subtropical areas that affects more than 12 million people worldwide [1]. Moreover, every year, 2 million new cases are diagnosed, among them, 75 % of the cases are cutaneous and 25 % are visceral leishmaniasis, leading to it being the second most common cause of parasite-associated death resulting in 20,000 to 30,000 deaths per year [2]. *Leishmania* preferentially infect phagocytic cells, as macrophages, neutrophils and dendritic cells of susceptible mammalian hosts [3] causing numerous clinical manifestations. In general, cutaneous leishmaniasis is located adjacent to the infectious site, the skin or lymph nodes. The parasite can escape into the nasal and oropharyngeal mucosa causing mucocutaneous leishmaniasis; or also migrate to the spleen, liver, bone marrow, and distant lymph nodes, leading to lethal clinical manifestations, named visceral leishmaniasis or kala-azar [4].

Leishmaniasis is transmitted by a female blood-sucking insects of the genus *Phlebotomus* in the 'Old' World and by species of *Lutzomya* in the 'New' World. The parasite can occur in two ways: the promastigote, which has high mobility, and is found in the digestive tract of the vector; and amastigote, without flagella, which develops into the phagolysosomes of phagocytic cells. Immediately before blood intake, the insect saliva containing promastigote forms is inoculated into the skin of the mammalian host. Soon after, the parasite is phagocytosed, remaining viable inside the phagolysosome, the fused phagosome and lysosome. Then, the promastigote form differentiates in amastigote approximately 12–24 h later [5, 6]. When an infected mammal host is bitten by the sand fly, it sucks amastigote-infected macrophages or free amastigotes which will transform into mobile flagellated promastigotes in the midgut of the vector. In this process, procyclic promastigotes (proliferative and non-infective forms) acquire the ability to be virulent and non-proliferative, the metacyclic promastigotes, and this process is called metacyclogenesis [7]. These promastigote forms migrate to the oral cavity promoting the transmission in the next blood meal.

The innate immune cells present in the skin are the first line of defense against *Leishmania* infection [8]. Dermal dendritic cells (DCs), Langerhans cells (LCs) [9, 10], mast cells, T cells, and macrophages are the immune cells in the skin. Interesting, keratinocytes, which are the most abundant in the skin, also play an active role in the local immune response and it has been reported that they have an important role in polarization of the Th1 response during leishmaniasis [11, 12]. After parasite inoculum into the dermis, neutrophils quickly infiltrate and phagocytose *Leishmania* parasites, becoming the first circulatory cells to reach tissue space [13–15]. Macrophages are the second wave of infiltrating immune cells and are the principal host cells for the *Leishmania* [16, 17]. Thus, neutrophils and macrophages play crucial roles in disease progression, but ironically as professional phagocytic and killing cells, they become targets because of evasion mechanisms employed by *Leishmania* to subvert the host immune system.

**Fig. 1:**
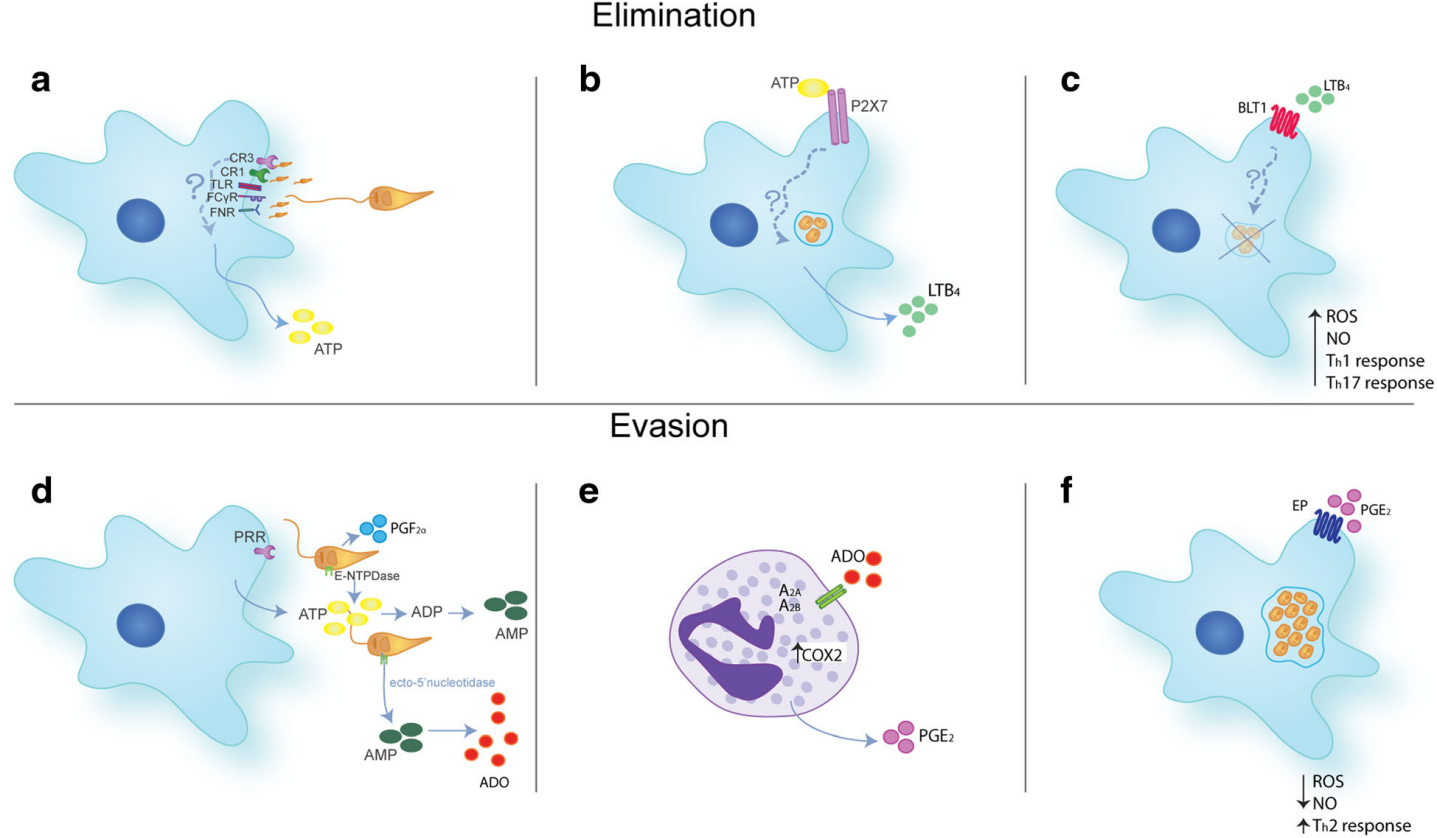
Schematic representation of elimination and evasion mechanisms mediated by purinergic signaling and lipid mediators during *Leishmania* infection. **a** *Leishmania* spp. promastigotes can be recognized by PRRs. This recognition leads to the release of ATP into the extracellular medium. **b** eATP active P2X7 receptors, which in turn leads to release of LTB_4_. **c** LTB_4_ binds to specific receptors on cell membrane, as BLT1, causing the elimination of *Leishmania* spp. by production of ROS, NO, and participates on T_h_1 and T_h_17 polarization. **d** In order to evade the immune system and ensure its survival, *Leishmania* spp. possess ecto-nucleotidase enzymes, such as E-NTPDase and ecto-5’-nucleotidase, removing eATP and favoring Ado accumulation. **e** Ado actives P1 receptors, such as A_2B_, increasing COX-2 expression and therefore leads to the release of PGE_2_. **f** PGE_2_ in turn binds EP receptors on cellular membranes, causing the decrease of ROS and NO production, and participates on T_h_2 polarization, resulting in establishment and dissemination of *Leishmania* spp*.* infection

Macrophages and neutrophils possess several pattern recognition receptors (PRR) that respond to pathogen-associated molecular patterns (PAMPs) present in the *Leishmania* surface, such as lipophosphoglycan (LPG) and glycoprotein 63 (GP63), both in humans and in mice [18–21]. Several host immune receptors can bind *Leishmania* components or antibodies against *Leishmania*, such as: the first and third complement receptor (CR1 and CR3, respectively) [22, 23], mannose receptor (MR) [24, 25], Fc gamma receptors (FcγRs) [26], fibronectin receptors (FNRS) [18], and Toll-like receptors (TLR) [27–30]. TLRs are phylogenetically the most ancient and best studied PRRs. In humans, 10 TLR family members have been identified and there are 12 in mice [31]. TLRs activation has been associated with the production and release of inflammatory mediators, such as cytokines, lipid mediators, and adenosine 5’-triphosphate (ATP) in extracellular medium [32–34]. ATP is widely present in the intracellular environment, at concentrations in the millimolar range, whereas it is almost imperceptible in the extracellular space, being around nanomolar [35]. We recently showed that *Leishmania amazonensis* recognition by macrophages leads to ATP release [36] (Fig. 1a). However, the receptor involved in this release and the mechanism that triggers this process has not yet been elucidated.

### Purinergic receptors

Extracellular ATP (eATP) is defined as a damage-associated molecular pattern (DAMP) causing biological effects though the activation of purinergic receptors that are presented on the cell membrane. Purinergic receptors are classified into two large families: P1 and P2. The P1 receptor family is characterized by metabotropic receptors activated by adenosine: A1, A2_a_, A2_b_, and A3 [37]. The P1 receptor activation has been discussed in several systems, suggesting a role in both physiological and pathological processes. In the immune system, P1 receptors are widely expressed by cells of the myeloid and lymphoid lineage [38]. P1 receptors act in regulating the immune response, and are involved mainly in resolving inflammation [39].

The P2 family of receptors is subdivided into P2X and P2Y. The P2Y receptors are G-protein coupled receptors, [35] while P2X are ionotropic receptors, capable of forming cationic channels activated by eATP. The participation of P2Y receptors in inflammatory events has been described [40, 41]. Furthermore, it has been reported that P2Y2 receptors act in neutrophil chemotaxis after activation by eATP [42]. The family of P2X receptors, in contrast, consists of ionotropic receptors. These receptors are intrinsic ion channels for Na^+^, K^+^, and Ca^2+^. To date, seven subtypes of P2X family have been cloned: P2X1 to P2X7 [43, 44]. The most studied of P2X receptors is the P2X7 subtype. This receptor has two transmembrane domains, being a polypeptide of 595 amino acids with a longer C-terminal domain, compared to other members of the P2X family. This peculiarity makes it capable of inducing the formation of pores permeable to molecules up to 900 Daltons after sustained eATP stimulation [45]. Moreover, the elongated C-terminal enables it to initiate various intracellular signaling cascades culminating with apoptosis, vesicular fusion, phospolipase D activation, exosome release, activation and secretion of pro-inflammatory cytokines IL-1β and IL-18 [44]. The expression of P2X7 receptor is well characterized under many cell types, including macrophages [46], monocytes [47], neutrophils [48], among others [49]. Furthermore, many studies have demonstrated the participation of purinergic receptors in the induction of bioactive lipid mediators [50–54].

### Lipid mediators

Lipid metabolites of arachidonic acid (AA), including leukotrienes (LTs) and prostaglandins (PGs), have emerged as important mediators of a variety of physiological and pathophysiological functions. They are synthesized through the metabolism of AA released by cytosolic phospholipase A_2_. The lipid metabolites can be subsequently metabolized by different pathways, including the cycloxygenase (COX) enzymes and lipoxygenase (LO) generating a range of bioactive eicosanoids, named PGs and LTs, respectively. The activation of cPLA_2_ and 5-LO involves an increase of intracellular Ca^2+^ and subsequently activation of certain protein kinases, as well as translocation of 5-LO from cytoplasm and nucleoplasm to membrane sites such as the nuclear envelope [55]. The AA is presented to 5-LO by an essential accessory protein called 5-LO activating protein (FLAP), producing an unstable precursor of all other leukotrienes, the LTA_4_ [55]. Once generated, LTA_4_ can be conjugated with reduced glutathione by LTC_4_ synthase (LTC_4_S) to form LTC_4_, or LTA_4_ can also be hydrolyzed by LTA_4_ hydrolase (LTA_4_H) to form LTB_4_ [56]. LTC_4_ as LTB_4_ can be exported to the extracellular space through specific transporters [57–59]. In the extracellular environment, LTC_4_ is rapidly converted to LTD_4_ by the glutamyl leukotrienase removing glutamic acid molecule of LTC_4_, and LTD_4_ can be further converted to LTE_4_ by the dipeptidase which removes a glycine residue of LTD_4_ molecule [60]. LTB_4_ is best known as a chemotactic and activator for leukocytes, and cysteinyl leukotrienes (LTC_4_, LTD_4_, and LTE_4_) are widely known in the pathogenesis of asthma [61].

PGs are formed when AA is metabolized by sequential actions of cyclooxygenase and their specific synthases [62]. COX has both cyclooxygenase (COX) and peroxidase activity, and three COX isoforms were described: COX-1, COX-2 and COX-3 [63, 64]. COX-1 and COX-3 are constitutively expressed while COX-2 is induced by inflammatory stimuli [64, 65]. There are six bioactive PGs: PGE_2_, PGI_2_, PGD_2_ and PGF_2_ [62]. Much is known about the pro-inflammatory functions of PGs, but, in the past years, it has been proven to also possess potential anti-inflammatory effects of PGs observed in resolution phase [66], and, importantly, these effects can be used by parasites to evade the immune system.

### Purinergic receptor, lipid mediators and immune evasion

The most effective mechanisms against infection by *Leishmania* already described involve the production of reactive oxygen species (ROS) and nitric oxide (NO) [67]. Furthermore, it has been shown that an effective response against infection by *Leishmania* is given by the induction of T_h_1 and T_h_17 responses [68–72], while T_h_2 response promotes susceptibility [68, 70].

The role of extracellular nucleotides and the activation of purinergic receptors during infection by *L. amazonensis* have been investigated [73]. Marques-da-Silva and colleagues [74] showed that P2Y2 and P2Y4 receptors have its expression upregulated and increased levels of uridine triphosphate (UTP) nucleotide into the extracellular environment during infection can lead to death of the macrophage by apoptosis and the elimination of the parasite. Other studies have shown that eATP can lead to the elimination of *L. amazonensis* in infected macrophages via P2X7 receptor [75]. A recent study demonstrates that elimination of *L. amazonensis* by P2X7 receptor depends on the production of LTB_4_ and leukotriene B_4_ receptor 1 (BLT1) [36] (Fig. 1b, c). Additionally, other studies have demonstrated the production of LTB_4_ in resistance to *L. amazonensis* and *L. braziliensis*, in humans and mice [76–78]. Furthermore, latest studies have demonstrated the participation of 15d-Prostaglandin J2 in *L. donovani* elimination [79]. This resistance can be due to the production of ROS and NO, which may be produced after P2X7 receptor activation [80, 81] and LTB_4_ release [82–85]. Moreover, the P2X7 receptor activation and LTB_4_ release have been implicated in the polarization of T_h_1 and T_h_17 responses, participating in the immune response against *Leishmania* [86–90] (Fig. 1c).

Regarding the participation of lipid mediators in *Leishmania* infection, the role of PGE_2_ in susceptibility has been discussed. It is known that PGE_2_ possesses anti-inflammatory activity, facilitating *Leishmania* infection in macrophages, suppressing inflammatory response in both cutaneous and visceral leishmaniasis [91–94]. Moreover, reinforcing the context of a beneficial effect of PGE_2_ for *Leishmania* survival, it was demonstrated that several *Leishmania* species possess lipid corpuscles as organelles and *L. infantum* is able to produce and release PGs, such as PGF_2α_ itself [95, 96] (Fig. 1d). It is important to highlight that PGE_2_ inhibits NO production [97], and T_h_1 and T_h_17 development [98–101] and, consequently, stimulates T_h_2 response, favoring infection [99] (Fig. 1f).

On the other hand, in order to perpetuate itself, *Leishmania* has developed methods to subvert microbicidal mechanisms and immune responses against itself. As already described before, eATP has proved to be an endogenous molecule able to induce the death of *L. amazonensis* through P2X7 receptors activation [36]. It has also been well established that the presence of enzymes capable of degrading ATP in the mammalian cell membrane forming ADP (adenosine-diphosphate) and adenosine (Ado), named ecto-nucleotidases. Among them, CD39 (ecto-NPTDase) and CD73 (ecto-5′-nucleotidase) exert relevant actions, regulating inflammatory responses of ATP and UTP. Thus, Ado is formed through the action of CD39 that converts ATP and ADP to 5'-adenosine mono-phosphate (AMP). AMP is the substrate for CD73. This enzyme, in turn, catalyzes the reaction that converts AMP to Ado [39, 102]. In this scenario, it has been shown that *Leishmania* express ecto-nucleotidase activity. This is confirmed by the observation of increased Ado levels in serum from visceral leishmaniasis patients [103, 104]. This can cause the prevention of the activation of macrophages and leads to the increase of infection by *Leishmania* species [105–109]. Moreover, the virulence of *L. amazonensis* promastigotes could be due to its high ecto-nucleotidase activity [110] (Fig. 1d). Moreover, ecto-5-nucleotidase activity also has been seen in *L. chagasi* [105]. Furthermore, it has been observed that *L. amazonensis* infection increases ecto-nucleotidases expression in DC [111]. Thus, the blocking of the A_2B_ receptors is found to increase production of NO and decrease parasite survival, suggesting participation of Ado in this process [109].

Others have shown that Ado increases COX-2 expression and PGE_2_ production in neutrophils [112, 113] (Fig. 1e). This corroborates the fact that both Ado and PGE_2_ stimulates the release of anti-inflammatory cytokines, such as interleukin (IL)-10 in macrophages [114, 115], while inhibiting the release of pro-inflammatory cytokines, such as tumor necrosis factor (TNF)-α and IL-12 in DCs and macrophages [116, 117]. This stimulates an anti-inflammatory environment, allowing establishment of infection.

It has been shown that Ado decreases production and release of LTB_4_ [118–121], which modulates microbicidal mechanisms. Moreover, it is known that *L. amazonensis* is capable to negatively modulate the production of LTB_4_ via P2X7 receptor activation in macrophages from C57BL/6 and BALB/c mice [36]. Neutrophils are recruited to the infection site when infection is initiated by sand fly bite [14, 122], spreading *Leishmania* parasites [17, 123, 124]. However, in other species of *Leishmania*, such as *L. braziliensis*, the neutrophils are important for parasite elimination [125]. Nevertheless, the role of the Ado in stimulation of PGE_2_ production in macrophages still needs to be studied. Moreover, other pathogens use Ado to subvert the immune system such as *Toxoplasma gondii*, *Staphylococcus aureus* and *Streptococcus agalactiae* [126–128].

The role of sand fly saliva substances in modulating *Leishmania* infection has been demonstrated [129, 130]. Furthermore, it has been described that sand fly saliva can inhibit NO production, and consequently increase the parasite load [131, 132]. It has also been described that *Lutzomyia longipalpis* saliva possesses ATPase activity, which can hydrolyse eATP [133]. Sand fly saliva also contains high levels of Ado, modulating the inflammatory micro-environment, causing NO inhibition, and macrophage inactivation, which in turn increases the parasitic load in macrophages and neutrophils [134–136]. Recently it was shown that exosomes are co-inoculated with *Leishmania* into mammalian hosts [137]. It is tempting to correlate it with a burst of ATP secretion, local Ado generation and PGE_2_ production. It is known that *L. longipalpis* saliva triggers the production and release of PGE_2_ and decreases LTB_4_ in macrophages [138, 139].

## Conclusion

**Fig. 2:**
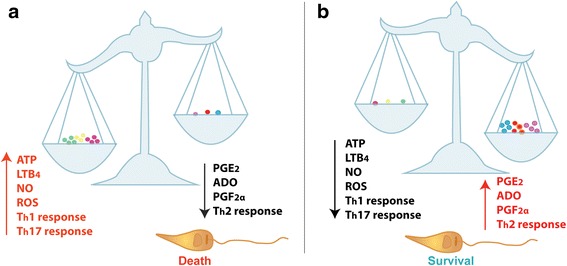
Balance between nucleotides and inflammatory lipid mediators on *Leishmania* spp. infection establishment. **a** The increase of ATP and reduction of Ado in extracellular medium leads to an overproduction of LTB_4_, which in turn stimulates the production of ROS and NO, and the polarization of immune responses for a T_h_1 and T_h_17 pattern; meanwhile a high PGE_2_ production also directs the polarization for T_h_2 response. This scenario facilitates the elimination of *Leishmania* spp. by macrophages. **b** The evasion of *Leishmania* spp. occurs when Ado concentrations in the extracellular medium overlaps the ATP. Thus, there is an increase in PGE_2_ and decreased ROS and NO, with consequent polarization T_h_2

The establishment of *Leishmania* infection can be due to the balance of several factors. Extracellular nucleotides can modulate the balance of pro- and anti-inflammatory factors such as PGs and LTs. To ensure their survival, *Leishmania* spp. developed strategies throughout its evolution to guarantee its perpetuation (Fig. 2a). The ability of *Leishmania* spp. to modulate extracellular concentrations of ATP and Ado, and consequently the balance of LTB_4_ and PGE_2_ shows how organisms can subvert the immune system of the host (Fig. 2b). Thus, the importance of knowledge of these strategies of evasion is essential in order to develop drugs capable to counterbalance *Leishmania* evasion.

## Abbreviations

ATP: 5′-adenosine triphosphate
eATP: extracellular ATP
Ado: Adenosine
DC: Dermal dendritic cells
LCs: Langerhans cells
LPG: Lipophosphoglyca
GP63: Glycoprotein 63
CR: Complement receptor
MR: Mannose receptor
FcγRs: Fc gamma receptors
FNRS: Fibronectin receptors
TLR: Toll-like receptor
DAMP: Damage-associated molecular pattern; cytosolic phospholipase A2
COX: Cicloxygenase
5-LO: 5-lipoxygenase
LTB_4_: Leukotriene B_4_
NO: Nitric oxide
ROS: Reactive oxygen species
PGE_2_: Prostaglandin E_2_
AMP: 5′-adenosine mono-phosphate
TNF: Tumor necrosis factor
UTP: Uridine triphosphate
PAMPs: Pathogen-associated molecular patterns
